# Combining brain-computer interfaces and multiplayer video games: an application based on c-VEPs

**DOI:** 10.3389/fnhum.2023.1227727

**Published:** 2023-08-03

**Authors:** Selene Moreno-Calderón, Víctor Martínez-Cagigal, Eduardo Santamaría-Vázquez, Sergio Pérez-Velasco, Diego Marcos-Martínez, Roberto Hornero

**Affiliations:** ^1^Biomedical Engineering Group (GIB), E.T.S Ingenieros de Telecomunicación, University of Valladolid, Valladolid, Spain; ^2^Centro de Investigación Biomédica en Red en Bioingeniería, Biomateriales y Nanomedicina (CIBER-BBN), Madrid, Spain

**Keywords:** brain-computer interfaces, code-modulated visual evoked potentials, video games, multiplayer, electroencephalography

## Abstract

**Introduction and objective:**

Video games are crucial to the entertainment industry, nonetheless they can be challenging to access for those with severe motor disabilities. Brain-computer interfaces (BCI) systems have the potential to help these individuals by allowing them to control video games using their brain signals. Furthermore, multiplayer BCI-based video games may provide valuable insights into how competitiveness or motivation affects the control of these interfaces. Despite the recent advancement in the development of code-modulated visual evoked potentials (c-VEPs) as control signals for high-performance BCIs, to the best of our knowledge, no studies have been conducted to develop a BCI-driven video game utilizing c-VEPs. However, c-VEPs could enhance user experience as an alternative method. Thus, the main goal of this work was to design, develop, and evaluate a version of the well-known ‘Connect 4' video game using a c-VEP-based BCI, allowing 2 users to compete by aligning 4 same-colored coins vertically, horizontally or diagonally.

**Methods:**

The proposed application consists of a multiplayer video game controlled by a real-time BCI system processing 2 electroencephalograms (EEGs) sequentially. To detect user intention, columns in which the coin can be placed was encoded with shifted versions of a pseudorandom binary code, following a traditional circular shifting c-VEP paradigm. To analyze the usability of our application, the experimental protocol comprised an evaluation session by 22 healthy users. Firstly, each user had to perform individual tasks. Afterward, users were matched and the application was used in competitive mode. This was done to assess the accuracy and speed of selection. On the other hand, qualitative data on satisfaction and usability were collected through questionnaires.

**Results:**

The average accuracy achieved was 93.74% ± 1.71%, using 5.25 seconds per selection. The questionnaires showed that users felt a minimal workload. Likewise, high satisfaction values were obtained, highlighting that the application was intuitive and responds quickly and smoothly.

**Conclusions:**

This c-VEP based multiplayer video game has reached suitable performance on 22 users, supported by high motivation and minimal workload. Consequently, compared to other versions of “Connect 4” that utilized different control signals, this version has exhibited superior performance.

## 1. Introduction

Video games allow users to interact with certain elements in a virtual environment. Given the pronounced progress made in recent years in this sector, it is currently estimated that 40% of the world's population plays video games, which equates to more than 3.1 billion consumers (DFC Intelligence, 2022). Studies support that playing video games in moderation can bring great advantages specially focused on improving cognitive skills (Granic et al., 2014; Reynaldo et al., 2021), such as increasing decision-making ability (Jordan and Dhamala, 2022), enhancing visual attention (Gan et al., 2020) and improving attention control (Anguera et al., 2013). Furthermore, studies indicate that the abstraction capacity caused by video games can reduce the sensation of pain (Raudenbush et al., 2009; Inan and Inal, 2019). In addition to these benefits, multiplayer video games also reflect the importance of social interaction (Obbink et al., 2012). Nevertheless, most video games are controlled by a keyboard, mouse or joystick, making accessibility difficult for people with severe motor disabilities. To address this, brain-computer interface (BCI) systems could serve as alternative technology to promote accessibility to video games (Kerous et al., 2018). For this reason, it seems appropriate to delve into the combination of video games and BCI systems, with the purpose of improving the quality of life and increasing the independence of people with certain disabilities.

A BCI is defined as a communication system that allows the user to interact with the environment without the involvement of muscles or peripheral nerves (Wolpaw et al., 2000). BCI systems allow the interpretation of user's intentions by monitoring and processing their brain activity. Although there are several techniques to record such activity, electroencephalography (EEG) is commonly used as it is non-invasive, portable and inexpensive in comparison with other methods. This procedure is performed by placing a set of electrodes on the scalp (Wolpaw et al., 2000).

In this context, controlling video games with BCI systems would not only help to promote their accessibility to users with motor disabilities, but could also allow exploring the influence of competitiveness, collaboration or motivation on brain dynamics. To contextualize these terms, it is worth remembering that within multiplayer video games there are two main modalities: collaborative and competitive. Collaborative video games are those in which two or more players must make decisions as a team to achieve a common purpose, whereas in a competitive game the participants are rivals who have to achieve a faster goal than their counterparts. In this sense, a previous study evaluated the influence on social interaction between participants when they played a cooperative game (Obbink et al., 2012), but not a competitive video game.

Several studies have proposed BCI-driven multiplayer video games. Luca et al. (2021) aimed to identify patterns of neuronal activity and connectivity through a neurofeedback (NF) video game featuring both competitive and collaborative modes. Another study, carried out by Bonnet et al. (2013), implemented a video game in which two users played a soccer game using sensorimotor rhythms (SMRs). This game included individual, collaborative and competitive modes, with an accuracy of 75.83%, 75.42%, and 74.58% respectively. The first two modes were tested on 20 users for comparison, while only 8 users participated in the competitive mode. Also, it should be noted scientific literature does not recommend these kind of endogenous in BCIs for communication and control due to allow the discrimination of small amount of classes, requiring more training and achieving lower accuracies. However, they are recommended for neurorrehabilitation purposes (Young et al., 2014; Cervera et al., 2018). In contrast, Grkk et al. (2013) developed a video game based on steady-state visual evoked potentials (SSVEPs) to evaluate social and cooperative interaction, although it did not include a competitive mode nor reported accuracy metrics. Also, they required the use of a mouse.

In addition, among all the multiplayer games transferred to BCI systems, two studies developed different versions of the famous competitive game “Connect 4” (Maby et al., 2012; Holz et al., 2013), which consists of lining up 4 coins of the same color on a vertical board. They used SMR and P300 evoked potentials, respectively. However, the results showed an accuracies of 62.25% (Holz et al., 2013) and 83.30% (Maby et al., 2012). Also, the evaluations were conducted on a small number of users, only 4 and 2 correspondingly, making it difficult to draw robust conclusions. The limitations of using these signals mentioned are mainly centered on several factors. On the one hand, in general, P300-based systems require a calibration phase, which typically lasts 20–30 min (Martnez-Cagigal et al., 2016). However, this time requirement increases significantly for SMRs, sometimes extending to hours or even days. Unfortunately, some individuals may never be able to learn to generate satisfactory results (Wolpaw et al., 2000). Furthermore, current state-of-the-art BCI systems have been demonstrated the accuracy and the selection speed of these systems is slower than other types of control signals.

Recently, code-modulated visual evoked potentials (c-VEPs) have been proposed as a novel control signal able to overcome the aforementioned limitations, achieving similar or even higher accuracy and selection speeds (Bin et al., 2009; Volosyak et al., 2020). This control signal encodes commands by utilizing shifted versions of a pseudo-random sequence. In this paradigm, referred to as circular shifting, a calibration template is computed based on the user's EEG response to the visual stimulation. Subsequently, the online decoding of the desired user's command becomes feasible by identifying the phase shift relative to the original template. BCI applications based on c-VEPs have achieved excellent performances both for healthy users (e.g., 90%) (Bin et al., 2009) and motor-disabled users (e.g., 79%) (Verbaarschot et al., 2021). As has been demonstrated to date, the use of c-VEPs holds great potential due to their ability to attain high accuracy with very short calibration and fast command selections (Bin et al., 2009). Despite the promising potential of c-VEP-based BCIs for communication and control, to the best of our knowledge, there are no BCI video game in the scientific literature utilizing this type of control signal. This knowledge gap motivates us to explore the viability of employing c-VEPs to control multiplayer BCI-based video games and assess their suitability for such applications.

The aim of this work is to design, develop and evaluate a multiplayer video game using c-VEPs, specifically, a version of the famous “Connect 4”, where two users compete to win. The application has beed evaluated by 22 healthy users during a single session, where three types of tasks were carried out: guided, free and competitive multiplayer. Finally, the usability of the application was assessed from two points of view: quantitative analysis, i.e., accuracy and speed; and qualitative analysis, i.e., satisfaction questionnaires. We will focus our attention in this game since it is an intuitive and well-known game, the matches are prompt and it allows evaluating of the competitiveness between two players reflecting a clear winner. In our proposed video game, users must attend to the position on the board where they wish to place a coin, while each available column's target cells is illuminated following an out-of-phase version of a pseudo-random sequence.

## 2. Subjects and methods

### 2.1. Subjects

A total of 22 healthy controls (aged 28 years ± 2.60 years, 10 females and 12 males) participated in the experiments. Six users had previous experience controlling BCI systems. All participants gave their informed consent and were informed in advance of the purpose of the study.

### 2.2. Stimulation paradigm and architecture of BCI system

**Figure 1:**
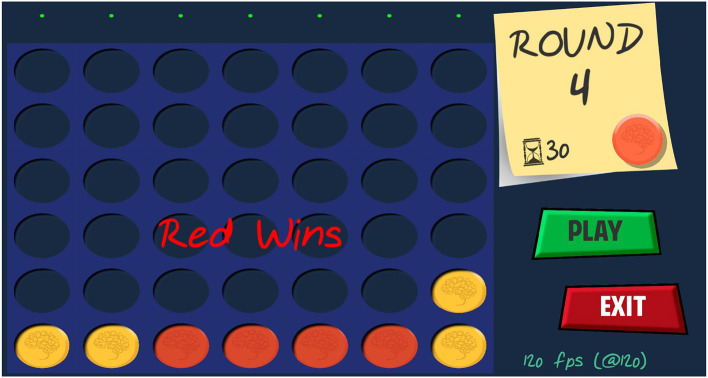
Snapshot of the graphical interface of the video game. The main board is located on the left side of the screen, while the current round and player are displayed on the right. In this case, the red player has won after lining up four coins horizontally.

Our “Connect 4” video game consists of a vertical board of seven columns and six rows. Two players, designated as player 1 and player 2, take turns placing their respective yellow and red coins. The objective of the game consist in aligning four coins of the same color horizontally, vertically or diagonally. Figure 1 shows a snapshot of the video game. To determine in which column the coin should fall at any given moment, a stimulation paradigm based on c-VEPs is used. Users must attend to the position on the board where they want to place a coin, while each of the target cells of columns available are illuminated following an out-of-phase version of a pseudo-random sequence (Martnez-Cagigal et al., 2021). Coin descends vertically, propelled by the force of gravity, so only one cell per column can be simultaneously flashing, except whenever a column is already filled. The user's attention to the visual stimulus generates a specific brain response in the EEG, allowing to differentiate the position where the user wants to place the coin by detecting the phase difference through a real-time signal processing pipeline (Martnez-Cagigal et al., 2021).

The code used to modulate the illuminations of the board commands corresponds to a binary maximum length sequence (m-sequence) of *N* = 63 bits, generated through a linear feedback shift register (LFSR) using the polynomial *x*^6^+*x*^5^+1 and initial state 110,000 (Holmes, 2007). Its autocorrelation function is flat; being 1 for the original signal and −1/*N* for the rest (Martnez-Cagigal et al., 2021). Since the refresh rate of the monitor allowed up to 144 Hz, the stimuli were presented at 120 Hz. Several studies have shown that the higher the frequency of stimulus presentation, the greater the user comfort (Gembler et al., 2018; Baaklar and Ider, 2019; Martnez-Cagigal et al., 2023). Therefore, a higher frequency has been opted for over the standard 60 Hz to improve system speed and enhance the satisfaction of users (Martnez-Cagigal et al., 2023). Thus, the duration of a complete cycle of the sequence corresponds to 0.525 s (i.e., 63/120). The selection matrix consists of seven commands, where each command controls each of the columns where a coin can be placed. Despite the flat autocorrelation of the sequence, the autocorrelation of the EEG response does not necessarily have to be so. Therefore it is desirable to space out the assigned delays as much as possible throughout the 63-bit code to facilitate its subsequent decoding (Martnez-Cagigal et al., 2021). In this case, the delays assigned to each column were set in multiples of 9 samples, i.e.; θ_*i*_ = 9·*i*, where *i* = 0, 1, ...6.

**Figure 2:**
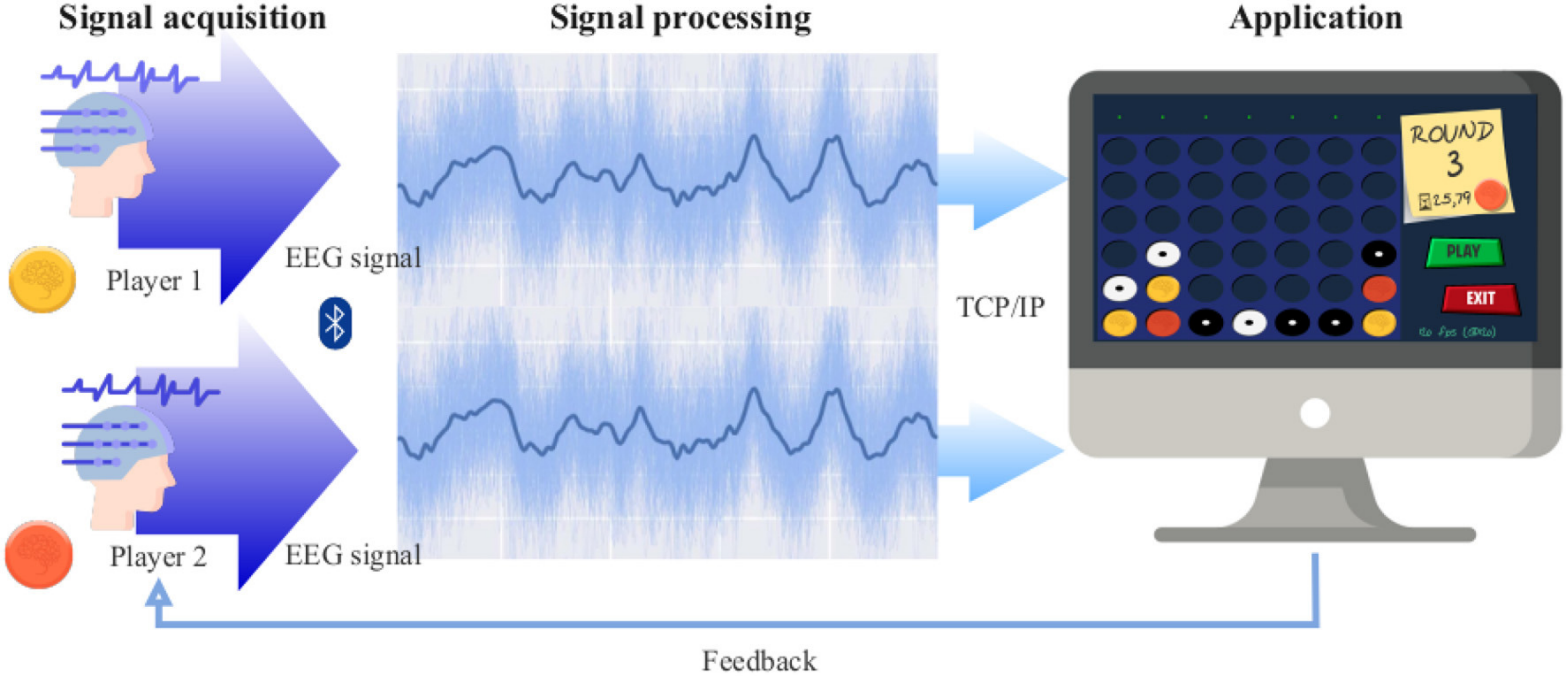
Architecture of BCI system, consisting of three stages: signal acquisition, where the EEG signal is recorded from the two participants; signal processing stage, where each EEG is processed (averaged EEG signals to the stimuli are also shown); and the application, the “Connect 4” video game in charge of giving real-time feedback to users.

As shown in Figure 2, the BCI system consists of 3 main stages: (1) signal acquisition; (2) signal processing; and (3) the video game application. These stages have been implemented within MEDUSA©, a software ecosystem for the development of BCI systems and neuroscience experiments www.medusabci.com (Santamara-Vazquez et al., 2023). The application has been specifically and entirely developed for this study, including the processing stages and graphical interface.

### 2.3. Signal acquisition

EEG signals were recorded using g.Nautilus Pro equipment (*g.Tec*, Guger Technologies, Austria), with a sampling rate of 250 Hz. EEgs were transmitted via Bluetooth to the computer running the video game instance. Eight gel-based electrodes were placed on the scalp at positions Fz, Cz, Pz, P3, P4, PO7, PO8, and Oz, using the right earlobe as reference (A2) and AFz as a ground, according to the International System 10-10 (Krusienski et al., 2008). The computer used was an Intel(R) Core(TM) i7-10700F CPU@ 2.90GHz, 32 GB RAM. Of note, the application was displayed in a Keep Out XGM24F+ 23.8" LED FullHD FreeSync monitor with a maximum refresh rate of 144 Hz.

#### 2.3.1. Processing stage

The first step in the processing stage consists of preprocessing the EEG signal to eliminate unnecessary frequency bands for c-VEP detection. The preprocessing consisted of a 7-order Butterworth infinite impulse response (IIR) bandpass filter with cutoff frequencies between 1 and 30 Hz. To decode the selected command in real-time, the standard processing method for c-VEPs based on circular shifting was used (Martnez-Cagigal et al., 2021). In this stage, two phases are distinguished: calibration and testing.

In calibration, the signal is recorded when the user looks at the command encoded with the original m-sequence (i.e., without delay) for *k* numbers of cycles (i.e., repetitions of the m-sequence). Two versions of the EEG response were obtained after preprocessing: (1) the concatenated epochs $A\in {\mathbb{R}}^{\left[kN_{s}\cdot N_{c}\right]}$ (i.e., *N*_*s*_ is the number of samples and *N*_*c*_ the number of channels) and (2) the epochs averaged over all cycles $B\in {\mathbb{R}}^{\left[N_{s}\cdot N_{c}\right]}$. To maximize the correlation between these two versions, canonical correlation analysis (CCA) was applied. The spatial filter *w*_*b*_ is selected as the projection that maximizes the correlation coefficient between *A* and *B*. Of note, the averaged version is replicated *k* times to match the dimensions of the concatenated version. Thereby, the main template was calculated by projecting the averaged response with the CCA-trained spatial filter. Templates for the rest of the commands were calculated by circularly shifting the main template according to each lag (Martnez-Cagigal et al., 2021). In addition, artifact rejection was applied, i.e., only non-noisy epochs were used to calibrate the system. The standard deviation (STD) of the signal was calculated for each channel. Then, an epoch was discarded if at least the STD in one channel exceeded three times the STD of that channel (Martnez-Cagigal et al., 2023).

Subsequently, in the test stage, the epochs of each trial are extracted and spatially projected with the spatial filter *w*_*b*_. Pearson's correlation coefficients of the projection with all templates are calculated. The command selected by the user will be the one corresponding to the delay whose template yielded the highest correlation (Martnez-Cagigal et al., 2021).

#### 2.3.2. Application

The application stage is in charge of interpreting the selected commands and providing feedback to the user in real time. This application consists of a board with seven columns and six rows where four coins of the same color must be aligned horizontally, vertically or diagonally. Initially, it is a competitive game, where one player controls the red coin and the other the yellow ones. This application can be used in both individual and competitive modes. In individual player mode, a single person manages the red and yellow coins, selecting them sequentially. If neither player manages to line up four coins vertically, horizontally or diagonally and there are no empty spaces on the board, the game would indicate that the players ended in a tie. Between one user and another, 3 s were given to think.

The graphical interface has been developed in Unity, a video game engine that uses C# programming language. Unity was selected because it enables control over the monitor refresh rate. For c-VEPs it is specially important to be precise in the stimulation times, since small jitter (i.e., latency variations) could lead to decoding mistakes (Martnez-Cagigal et al., 2021). The communication between the graphical interface and the processing stage was carried out using an asynchronous TCP/IP client-server architecture.

On the other hand, it is worth mentioning that much of the research in the field of BCI and video games are focused on the technical challenges of these systems, such as improving real-time processing or accuracy. However, little attention is paid to the final application, despite previous studies suggesting its relation to system performance (Nijboer et al., 2010). Examples of such factors are developing user-friendly graphical interfaces or using control paradigms more in line with end-user characteristics. Taking this into account, our intention was to develop a visually appealing and dynamic application that showcases the current round number, identifies the active player, and tracks the elapsed time. To minimize distractions from adjacent stimuli and enhance the accuracy of command selection, a dot has been incorporated within each cell.

To promote open science, the application has been developed as a MEDUSA© Platform app, being available in the app market www.medusabci.com/market/connect4/ (Santamara-Vazquez et al., 2023).

### 2.4. Experimental protocol

In order to evaluate the usability of the application, a mixed analysis was performed. Firstly, a quantitative analysis, measuring accuracy and speed-related parameters such as output characters per minute (OCM) and information transfer rate (ITR). The accuracy of all tasks was calculated for each user, considering the number of correct selections and the number of selections made. A correct selection was considered when the coin fell to the position corresponding to the cell where the user was looking at.

Although the ITR is the most widely used metric, studies suggest that it relies on assumptions that are often inaccurate in online BCI systems (Speier et al., 2013; Yuan et al., 2013). The ITR was calculated through the following formula:


(1) $$\begin{array}{l} ITR\left(\frac{bits}{min}\right)=Q\left(log_{2}\left(S\right)+P\cdot log_{2}\left(P\right)+\left(1-P\right)log_{2}\left(\frac{\left(1-P\right)}{\left(S-1\right)}\right)\right) \end{array}$$

where *Q* represents the number of selections per minute, *P* is the accuracy and *S* is the number of commands. The number of selections per minute was 11.43, calculated from the selection time per command, which was 5.25 s. This time was calculated without considering the pause times, taking into account that 10 cycles were used, with each cycle lasting 0.525 s. Additionally, the accuracy was calculated based on the number of correct selections out of the total number of selections made. Lastly, the number of commands was 7, based on the number of columns. Therefore, we will also calculate the OCM. This is computed by dividing the total number of selections by the time required to select them in minutes. This metric is specially useful for estimating the system communication rate in asynchronous systems (Speier et al., 2013). Additionally, a qualitative analysis was carried out by means of questionnaires such as system usability scale (SUS) and NASA task load index (NASA-TLX) (Hart and Staveland, 1988).

As for the number of sessions, one session was conducted per user. Each session consisted of a series of tasks that lasted 45 min in total. The performance of these tasks was divided into two parts: individual and competitive assessment. Once the user had completed their individual tasks, they were paired with another person to carry out the competitive multiplayer mode.

Prior to the completion of all the tasks, a calibration was conducted to determine the user's c-VEP templates and required to identify where the user was looking at any given moment. In this calibration, the user was asked to focus their attention on the first column (corresponding to the command that encodes the original m-sequence, without lag). Ten trials of 10 cycles each were recorded, therefore the calibration stage lasted 52.50 s per user. Subsequently, a decoding model was trained following the signal processing pipeline detailed in Section 2.3.1.

Upon completion of the calibration, the next steps were to perform the tasks. Each command had a selection time of 5.25 s, equivalent to 10 established cycles. Firstly, individual tasks were performed. These were divided into guided and free tasks, with the same user controlling both the yellow and red coins.

- Guided tasks. The objective was to arrange the coins in a specific manner as instructed by the supervisor. Four tasks were performed, requiring 15, 17, 21, and 25 selections, respectively.
- Free tasks. The objective was to win the match freely, with some autonomy. It did not matter in which row or column the coins were positioned as long as they were aligned with the color and the way they were previously specified (e.g., placing yellow coins vertically). Three tasks were carried out.

It is important to note that in cases where a user accidentally selected the wrong command, they were required to report it, resulting in an increase in the total number of final selections. Once the individual tasks were completed, the competitive assessment was carried out through multiplayer tasks:

- Multiplayer tasks. Three matches were played in which two users competed to win. For this purpose, two EEG signals were recorded in parallel, each with its own processing pipeline. A different player started each match.

After carrying out all the tasks, participants were asked to fulfill standardized NASA-TLX and SUS questionnaires to assess their workload and satisfaction. NASA-TLX is one of the most widely used methods for assessing mental workload and fatigue. This procedure gives an overall workload score based on a weighted average of the scores of 6 subscales ( i.e., mental demand, physical demand, temporal demand, performance, effort and frustration), defining the relevant factors in the user's subjective experience of workload (Hart and Staveland, 1988). On the other hand, satisfaction was assessed through a SUS questionnaire, which offered an overview of the usability of the application, alternating 10 positive and negative questions about the application to avoid acquiescence bias (Brooke, 1995). The questionnaire was based on a 5-point Likert scale (Likert, 1932). Additionally, the last question focused on collecting suggestions to improve the application.

## 3. Results

### 3.1. Quantitative analysis

Table 1 details the average accuracies and ITRs of each user for each type of task, as well as for the sum of all of them. The average accuracy for guided, free and multiplayer tasks was 94.39%, 95.43%, and 91.40%, respectively. The global average was 93.74% ± 1.71%. In terms of system speed-related parameters, an ITR of 30.61 bpm ± 0.34 bpm was reached. The results of the OCM for different number of cycles in guided tasks are presented in Table 2. As it can be observed, the OCM for 10 cycles was 11.43 selections per minute. The table also displays the accuracy for all users for each cycle, highlighting that some users reached their peak accuracy using fewer cycles, resulting in a shorter selection time.

**Table 1:** Quantitative results of the assessment by all users for each set of tasks, as well as the overall average.

**User**	**Guided tasks**		**Free tasks**		**Multiplayer tasks**		**Average**	
	**Accuracy**	**ITR (bpm)**	**Accuracy**	**ITR (bpm)**	**Accuracy**	**ITR (bpm)**	**Accuracy + STD**	**ITR + STD**
U01	98.75%	31.73	100.00%	32.08	100.00%	32.08	99.58% ± 0.59%	31.96 ± 0.16
U02	96.25%	31.14	93.33%	30.52	95.23%	30.91	94.93% ± 1.21%	30.85 ± 0.25
U03	91.66%	30.19	93.33%	30.52	90.50%	29.98	91.83% ± 1.16%	30.23 ± 0.22
U04	95.24%	30.91	96.43%	31.18	87.03%	29.36	92.90% ± 4.18%	30.48 ± 0.80
U05	100.00%	32.08	100.00%	32.08	85.00%	29.02	95.00% ± 7.07%	31.06 ± 1.44
U06	85.33%	29.08	86.67%	29.30	88.33%	29.5	86.77% ± 1.22%	29.32 ± 0.20
U07	92.13%	30.29	92.85%	30.43	91.53%	30.17	92.17% ± 0.54%	30.29 ± 0.11
U08	91.95%	30.25	88.63%	29.64	100.00%	32.08	93.52% ± 4.77%	30.65 ± 1.03
U09	97.56%	31.43	100.00%	32.08	100.00%	32.08	99.18% ± 1.15%	31.86 ± 0.31
U10	78.48%	28.04	81.08%	28.41	71.20%	27.11	76.91% ± 4.17%	27.85 ± 0.54
U11	100.00%	32.08	100.00%	32.08	100.00%	32.08	100.00% ± 0.00%	32.08 ± 0.00
U12	100.00%	32.08	100.00%	32.08	95.23%	30.91	98.41% ± 2.25%	31.69 ± 0.55
U13	100.00%	32.08	100.00%	32.08	86.57%	29.28	95.52% ± 6.33%	31.14 ± 1.32
U14	100.00%	32.08	100.00%	32.08	86.06%	29.19	95.35% ± 6.57%	31.11 ± 1.36
U15	98.76%	31.73	100.00%	32.08	95.23%	30.91	97.99% ± 2.02%	31.57 ± 0.49
U16	91.86%	30.23	96.43%	31.18	85.94%	29.18	91.41% ± 4.29%	30.19 ± 0.81
U17	96.43%	31.18	100.00%	32.08	100.00%	32.08	98.81% ± 1.68%	31.78 ± 0.42
U18	86.75%	29.31	84.22%	28.89	100.00%	32.08	90.32% ± 6.19%	32.09 ± 1.41
U19	88.06%	29.54	90.00%	29.88	96.29%	31.14	91.45% ±3.51%	30.18 ± 0.69
U20	88.63%	29.64	96.42%	31.17	96.66%	31.23	93.90% ± 3.73%	30.68 ± 0.73
U21	100.00%	32.08	100.00%	32.08	77.81%	27.94	92.60% ± 0.59%	30.70 ± 0.16
U22	98.71%	31.71	100.00%	32.08	82.14%	28.57	93.61% ± 8.13%	30.78 ± 1.57
**Average**	**94.39%**	**30.85 bpm**	**95.43%**	**31.09 bpm**	**91.40%**	**30.31 bpm**	**93.74%** ±**1.71%**	**30.61 bpm** ±**0.34 bpm**
**STD**	**5.86%**	**1.16 bpm**	**5.77%**	**1.18 bpm**	**7.89%**	**1.46 bpm**		

ITR, information transfer rate; STD, standard deviation.

**Table 2:** Performance metrics of each user in function of the number of cycles for guided tasks.

**No. Cycles**	**1**	**2**	**3**	**4**	**5**	**6**	**7**	**8**	**9**	**10**
**Time (s)**	**0.53**	**1.05**	**1.58**	**2.10**	**2.62**	**3.15**	**3.68**	**4.20**	**4.73**	**5.25**
**OCM (select/min)**	**113.21**	**57.14**	**37.97**	**28.57**	**22.90**	**19.05**	**16.30**	**14.28**	**12.68**	**11.43**
U01	26.25	47.50	67.50	73.75	87.50	93.75	97.50	96.25	97.50	**98.75**
U02	23.75	47.50	60.00	76.25	81.25	85.00	88.75	92.50	91.25	**96.25**
U03	17.85	34.52	46.43	51.19	66.66	67.85	76.19	79.76	84.52	**91.66**
U04	17.85	34.52	54.76	60.71	78.57	85.57	83.33	85.71	91.66	**95.24**
U05	33.33	71.79	93.59	98.71	**100.00**	100.00	100.00	100.00	100.00	100.00
U06	26.66	36.00	44.00	45.33	60.00	68.00	68.00	74.66	77.33	**85.33**
U07	25.84	41.57	51.68	64.04	69.66	76.40	80.89	85.39	86.51	**92.13**
U08	21.84	29.88	54.02	58.62	63.22	64.36	70.11	74.71	80.46	**91.95**
U09	31.70	52.44	69.51	73.17	80.48	85.36	87.80	90.24	93.90	**97.56**
U10	17.72	22.78	36.71	49.36	53.16	55.69	59.49	65.82	72.15	**78.48**
U11	37.18	58.97	79.48	91.02	92.30	97.43	**100.00**	100.00	100.00	100.00
U12	28.75	65.00	77.5	91.25	95.00	97.50	98.75	98.75	**100.00**	100.00
U13	35.04	67.53	85.71	94.80	96.10	96.10	98.70	98.70	**100.00**	100.00
U14	24.36	46.15	61.54	80.77	87.18	96.15	96.15	98.71	**100.00**	100.00
U15	29.63	49.38	66.66	81.48	85.18	88.88	91.35	95.06	95.06	**98.76**
U16	26.74	34.88	40.69	47.67	62.79	70.93	77.91	84.88	**91.86**	91.86
U17	15.47	41.66	55.95	66.66	76.19	83.33	85.71	89.28	91.66	**96.43**
U18	18.07	25.30	32.53	44.58	44.58	55.42	68.67	72.29	75.90	**86.75**
U19	26.86	34.33	38.80	53.73	64.18	64.18	73.13	77.61	80.59	**88.06**
U20	22.72	37.50	43.18	61.36	64.77	64.77	65.91	72.72	81.81	**88.63**
U21	33.33	55.13	74.36	84.61	89.75	94.87	98.71	97.43	**100.00**	100.00
U22	34.61	60.25	67.95	80.77	85.89	91.02	92.31	94.87	**98.71**	98.71
**Average**	**26.17**	**45.21**	**59.21**	**69.53**	**76.56**	**81.02**	**84.51**	**87.51**	**94.49**	**94.39**

OCM: output characters per minute. Maximum accuracy achieved is shown in bold.

### 3.2. Qualitative analysis

**Figure 3:**
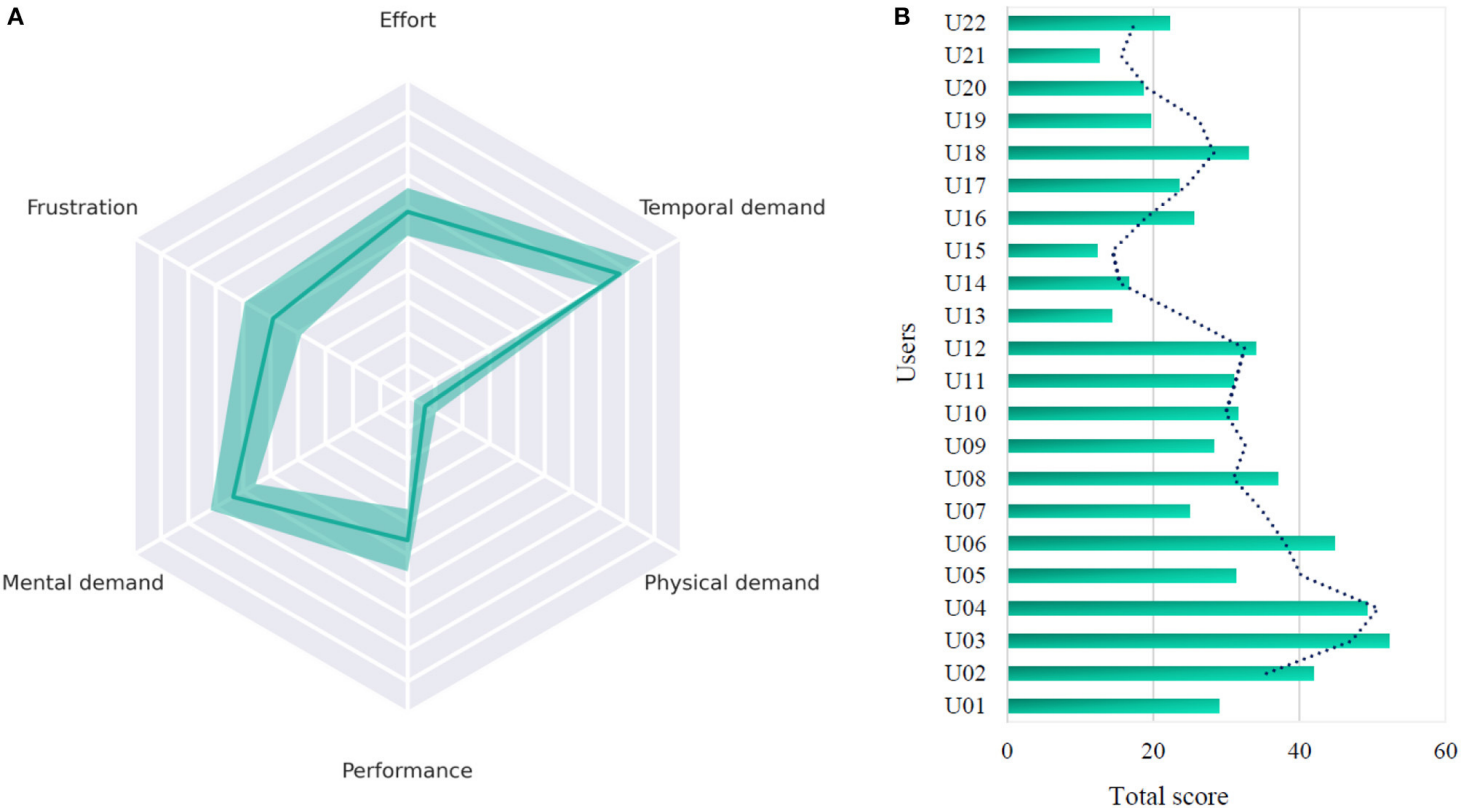
Results of the NASA-TLX questionnaire. **(A)** Distribution of the variables that contributed the greatest workload for all users. **(B)** Overall score for each user.

NASA-TLX questionnaire carried out by the users indicates a low workload (total of 28.82 ± 11.10 points out of 100), calculated as the weighted average of the scores for the 6 parameters (Hart and Staveland, 1988). The total workload score for each user as well as the variables that are most relevant after the use of the application for all participants are displayed in Figure 3.

SUS questionnaire results are shown in Table 3. The statements were evaluated between 1 (strongly disagree) and 5 (strongly agree) points. All positive statements (odd numbering) were rated between 4 and 5 points, and most of the negative ones (even numbering) were rated below 3 points. It is noteworthy to mention that users were considerably satisfied with the BCI video game, and a high satisfaction value was obtained (SUS coefficient of 80.60 out of 100) (Brooke, 2013). According to the results, the application is intuitive, easy to use and responds with fluidity. Regarding the open-ended question, some suggestions for improvement were indicated, such as increasing the spacing between illuminations and giving more time between a selection and the onset of the following stimulation in order to plan the next move. On the other hand, some users reported feeling confident making a selection in less time than proposed.

**Table 3:** Results of questionnaire SUS for all users.

	**Statements**	**Mean + STD**
1.	The application is intuitive and easy to understand.	5.00 ± 0.00
2.	The application requires too much concentration.	3.18 ± 0.98
3.	I would imagine that most users would learn to use this application quickly.	4.86 ± 0.34
4.	The duration of the assessment session was too long.	1.86 ± 0.75
5.	The session is entertaining.	4.77 ± 0.42
6.	I had difficulty selecting the desired commands.	1.95 ± 0.97
7.	The application responds quickly and smoothly.	4.90 ± 0.28
8.	The time needed to select a command does not seem to be adequate.	2.90 ± 1.38
9.	I would like to use this application frequently.	4.41 ± 0.71
10.	The flickering stimuli of the application's cells are annoying.	1.90 ± 0.79

Average score of each statement evaluated between 1 and 5 points.

## 4. Discussion

### 4.1. Quantitative analysis

As can be seen in Table 1, high accuracy has been achieved for all tasks (>91%), thus verifying its correct functioning, since a BCI system is considered to be controllable when the user achieves more than 70% accuracy (Martnez-Cagigal et al., 2016). A Wilcoxon signed-rank test was used to perform the statistical analysis, although no significant differences in accuracy were found between tasks (*p*-values > 0.05), i.e., this study found that competitiveness did not have a significant impact on performance, with all users achieving high accuracy. However, it is worth noting that the accuracy for multiplayer tasks is slightly lower than for the other tasks. In this regard, a higher accuracy in the multiplayer tasks could be expected due to a higher motivation and stimulates effort and the desire for self-improvement. Nonetheless, it has been demonstrated that playing against a person results in higher arousal levels compared to playing against a computer (Ravaja et al., 2006). Furthermore, several studies have demonstrated that heightened fatigue and diminished arousal levels can give rise to attention deficits, increased workload, and lower performance (Kthner et al., 2014; Saha et al., 2021). In this case, this increased arousal may cause fatigue or pressure due to the competition, leading to a loss of concentration which can be reflected negatively in the results. Thus, this pressure and low concentration could prevail over the added motivation.

On the other hand, the reached ITR (30.61 bpm ± 0.34 bpm) could seem low compared to other c-VEP-based applications (Martnez-Cagigal et al., 2021). This is due the low number of possible commands (i.e., 7 columns), which is directly reflected in the ITR calculation. For this reason, the command selection time could be a more relevant metric to characterize the speed of the system, which in this case has been 5.25 s per command. A detailed evolution of the accuracy and selection time for the set of guided tasks as a function of the number of cycles used is shown in Table 2. It can be observed that several users would have been able to reach the maximum accuracy with a lower number of cycles than the established. For instance, user U05 could have reached the maximum accuracy with five cycles, which would be equivalent to 2.62 s per selection. On average, the number of cycles required to reach maximum accuracy per user was 9.36, as many users required 10 cycles.

### 4.2. Qualitative analysis

#### 4.2.1. NASA-TLX

Figure 3A provides the importance of each of the 6 NASA-TLX variables as a cause of the workload and their importance in each task for all users. The ratings for frustration, performance and physical demand are remarkably low. The parameter that affected most the experiment was the temporal demand, i.e., some users felt certain time pressure due to the pace of command selection. It could be deduced that workload could influence the decrease in performance. Specifically, 6 participants experienced a drop in accuracy during competitive tasks. Unlike the individual mode, where the intended coin placement was known, the competitive mode required the participants to devise their own strategy and account for their opponent's move in just 5.25 seconds. This might caused a loss of confidence and reduced focus on the stimulus presentation.

Figure 3B shows that most users had a low score on the NASA-TLX assessment (with an overall score of 28.82 out of 100). A high NASA-TLX value means a higher workload. For instance, users U03 and U04 reported the two highest NASA-TLX scores (52.3 and 49.3 respectively), but even so obtained an accuracy of 91.83% and 92.90% respectively. Hence, it is our belief that mental workload is not a problem in our system, but rather an inherent aspect of BCI systems. In general, users emphasized that they did not experience too much fatigue, so this allowed them to use the application for a lengthy period of time without experiencing eyestrain. On the other hand, users felt confident and relaxed as they made the selections and indicated that the proposed tasks were easy and straightforward, and that they were satisfied with the level of performance. After applying Spearman's Rank-Order correlation between the questionnaire values and the total accuracies for each user, a correlation of -0.26 was found and was not statistically significant (*p*-value > 0.05), i.e., a weak correlation was found between high NASA-TLX scores and decreasing accuracies.

#### 4.2.2. Questionnaire SUS

Results of the questionnaire SUS are depicted in Table 3. As can be seen, in terms of qualitative analysis, the questionnaire reflects that the developed application complies with the requirements and achieves high satisfaction values. The first statement reached the highest value, indicating that the developed application is accessible and convenient. Besides, all positive statements reached values above the mean (>4.4). On the other hand, in terms of negative statements, users reported that the application required a significant level of concentration, which could be attributed to the need to make a strategy before on making a move against the other user before the lighting sequence began. This was because the users did not have much time between selections (only 3 s until the illumination appeared). The most discordant responses came from the difficulty of command selection, which is closely related to the user's accuracy. In general, the greater the perceived difficulty, the lower accuracy obtained. Another parameter to be highlighted was the selection time. Mostly, participants that yielded low accuracy suggested to increase the inter-stimulus interval to have more time to think, whereas those who completed the task with nearly zero errors thought they were able to make faster selections. Moreover, the former also indicated that they found the stimulation annoying.

### 4.3. Comparison with previous studies

The applications with further similarities to our application were those of Holz et al. (2013) and Maby et al. (2012) since they implemented versions of the ‘Connect 4' game with other control signals. Table 4 summarizes the main differences between these and our study.

**Table 4:** Summary of comparison with other ‘Connect 4' video game versions.

**Study**	**Year**	**Control signal**	**N°Users**	**Multiplayer**	**Processing stage**	**Accuracy + STD**	**Selection time**
Holz et al. (2013)	2013	SMR	4	No	rLDA	62.25% ± 10.75%	15 s
Maby et al. (2012)	2012	P300	2	Yes	xDawn algorithm + Bay.	83.30% ± 5.18%	4.06 s
Present study	2023	c-VEPs	22	Yes	CCA	93.74% ± 1.71%	5.25 s

rLDA: linear discriminant analysis with shringake regularization, CCA: canonical correlation analysis, SMR: sensorimotor rhythm, c-VEPs: code-modulated visual evoked potentials, Bay: Bayesian classifier, STD: standard deviation.

First of all, concerning the number of users evaluated, in the study by Holz et al. (2013), their application was assessed in 4 users with severe motor restrictions. On the other hand, in the study by Maby et al. (2012), the experimental evaluation was performed with two control users. A larger number of users would be desirable to reach conclusive results. In our study, 22 control users were evaluated (10 females and 12 males), providing more heterogeneity and improving the statistical power.

In terms of the type of control signal used, Holz et al. (2013) used the elicited SMRs via hand and foot motor imagery (MI). SMRs are endogenous signals produced by imagining or performing limb movements. SMR-based BCI systems have been applied for rehabilitation due to could cause neuronal plasticity as an endogenous signal. However, SMRs are not an optimal selection in BCI systems for communication and control, since only 2-4 classes can be decoded and it takes a lot of time to train, not to mention that some people do not generate it properly. Often, they also get lower accuracies than for P300, SSVEP and c-VEPs (Martnez-Cagigal et al., 2016). In this case, an average accuracy of 62.65% was obtained with an average ITR of 0.53 bpm. On the other hand, Maby et al. (Maby et al., 2012) used P300 evoked potentials. P300 have been widely used in communication and control BCI applications as they allow to reach high accuracies with moderate selection times (e.g., >90%, 10–25 bpm) with a calibration of 20–30 min (Martnez-Cagigal et al., 2016). In this case, they obtained an average performance of 83.30% at 37.00 bpm. In this sense, the accuracy of our study (93.74% ± 1.71%) was significantly higher than the reported by Maby et al. (2012) (*p*-value=0.004) and the reported by Holz et al. (2013) (*p*-value=0.00026) after applying Mann-Whitney U-Test. With regard to the speed of the system, the ITR value for Holz et al. (2013) was of 1.44 bpm. However, in the case of Maby et al. (2012) it was 37.01 bpm, slightly higher than the one obtained for our application (30.61 bpm).

Concerning the selection time, Holz et al. (2013) used 15 s per selection, while Maby et al. (2012) required a theoretical selection time of 4.06 s, which resulted in a real 16.46 s since users were given 10 s to think before blinking. In our study, the theoretical selection time was 5.25 s after using 10 cycles with very high accuracies for all users. This short selection time could reduce the possible occurrence of fatigue with respect to the time required in the others studies like in Holz et al. (2013). Furthermore, each user was given 3 s to think before the illumination appeared. Also, as we have observed before, some users obtained an accuracy of 100% with only five and seven cycles, which would be equivalent to a selection time of 2.62 s and 3.68 s, respectively. In most studies that utilize P300 as a control signal, a longer time per selection is necessary due to the use of more sequences to enhance decoding accuracy (they used only 2).

Another parameter to note is the calibration time. The c-VEPs do not require an extensive calibration (Martnez-Cagigal et al., 2021) (52.50 s were used in our protocol). In the cases of the “Connect 4” adaptations, Holz et al. (2013) did not require a calibration stage, but an endogenous training stage that lasted between 26.26 min and 3 h, depending on the user. Maby et al. (2012) used a calibration that lasted 4.26 min, corresponding to 63 selections with two sequences per trial (repetitions of the same column flashing).

Regarding qualitative aspects, our workload assessed by the NASA-TLX questionnaire for all users was 28.82 points, whereas Holz et al. (2013) obtained 37.75 points. They also reported moderate workload and frustration, leading to fatigue for some participants. Maby et al. (2012), by contrast, did not evaluate any fatigue-related parameters.

### 4.4. Limitations and future work

Despite the satisfactory results and the successful achievement of the objective, several limitations were identified. Firstly, although the effectiveness of the BCI video game developed with healthy users has been demonstrated, we believe that it is necessary to evaluate the application with people with severe motor disabilities, since they have traditionally been the target application of BCI systems for communication and control. On the other hand, in order to achieve enhanced user comfort and greater ease of use, the exploration of dry electrodes could be considered as they do not require a conductive gel, thereby eliminating the need for its application and subsequent cleaning. In line to improve user comfort, it would also be advisable to use non-binary sequences (Gembler et al., 2018) or “amplitude depth reduction” (Ladouce et al., 2022) to reduce visual fatigue caused by the stimulus.

Similarly, in terms of technical aspects, it would be advisable to apply ‘early stopping' techniques. These algorithm would dynamically detect the number of cycles necessary to issue a selection, so it would not be necessary to wait for the 10 established cycles to make the decision. Some users felt that they could control the system with a lower number of cycles, which could have lead to a drastical ITR and speed increase. On the other hand, it would be advisable to develop an asynchronous stage (i.e., non-control detection) to monitor users' attention. In this way, users could think about the strategy to follow and voluntarily activate the system when they are ready.

## 5. Conclusion

This study focused on designing, developing and evaluating a version of the popular multiplayer game “Connect 4” with a BCI system based on c-VEP. The application was evaluated on 22 healthy users, obtaining promising results. An average accuracy of 93.74% ± 1.71% was achieved, suggesting that the use of c-VEPs is appropriate for developing a multiplayer competitive video game. These results were obtained with a calibration time of 52.5 s per user. It should also be noted that the command selection time was 5.25 s. On the other hand, the satisfaction questionnaire completed by the different participants suggests that the developed application is intuitive, easy to use and responds with speed and fluency. They also indicated low values of frustration and effort.

Furthermore, in comparison with two developments of the same video game that used SMR and P300 as control signals (Maby et al., 2012; Holz et al., 2013), our study achieved superior quantitative and qualitative results, thus demonstrating the favorable performance of the developed application based on c-VEPs.

## Data availability statement

The raw data supporting the conclusions of this article will be made available by the authors, without undue reservation.

## Ethics statement

Ethical review and approval was not required for the study on human participants in accordance with the local legislation and institutional requirements. The patients/participants provided their written informed consent to participate in this study.

## Author contributions

SM-C: contributed to conceptualization, methodology, software, validation, formal analysis, investigation, data curation, writing—original draft, writing—review and editing, and visualization. VM-C: methodology, software, validation, and writing—review and editing. ES-V: software and writing—review and editing. SP-V and DM-M: writing—review and editing. RH: writing—review and editing, supervision, project administration, and funding acquisition. All authors contributed to the article and approved the submitted version.
